# Optimization of End-to-End Convolutional Neural Networks for Analysis of Out-of-Hospital Cardiac Arrest Rhythms during Cardiopulmonary Resuscitation

**DOI:** 10.3390/s21124105

**Published:** 2021-06-15

**Authors:** Irena Jekova, Vessela Krasteva

**Affiliations:** Institute of Biophysics and Biomedical Engineering, Bulgarian Academy of Sciences, Acad. G. Bonchev Str. Bl 105, 1113 Sofia, Bulgaria; irena@biomed.bas.bg

**Keywords:** deep learning, deep neural network (DNN), electrocardiogram (ECG), cardiopulmonary resuscitation (CPR), chest compressions, ventricular fibrillation (VF), out-of-hospital cardiac arrest (OHCA), shock advisory decision, feature extraction, automated external defibrillator (AED)

## Abstract

High performance of the shock advisory analysis of the electrocardiogram (ECG) during cardiopulmonary resuscitation (CPR) in out-of-hospital cardiac arrest (OHCA) is important for better management of the resuscitation protocol. It should provide fewer interruptions of chest compressions (CC) for non-shockable organized rhythms (OR) and Asystole, or prompt CC stopping for early treatment of shockable ventricular fibrillation (VF). Major disturbing factors are strong CC artifacts corrupting raw ECG, which we aimed to analyze with optimized end-to-end convolutional neural network (CNN) without pre-filtering or additional sensors. The hyperparameter random search of 1500 CNN models with 2–7 convolutional layers, 5–50 filters and 5–100 kernel sizes was done on large databases from independent OHCA interventions for training (3001 samples) and validation (2528 samples). The best model, named CNN3-CC-ECG network with three convolutional layers (filters@kernels: 5@5,25@20,50@20) presented Sensitivity Se(VF) = 89%(268/301), Specificity Sp(OR) = 91.7%(1504/1640), Sp(Asystole) = 91.1%(3325/3650) on an independent test OHCA database. CNN3-CC-ECG’s ability to effectively extract features from raw ECG signals during CPR was comprehensively demonstrated, and the dependency on the CPR corruption level in ECG was tested. We denoted a significant drop of Se(VF) = 74.2% and Sp(OR) = 84.6% in very strong CPR artifacts with a signal-to-noise ratio of SNR < −9 dB, *p* < 0.05. Otherwise, for strong, moderate and weak CC artifacts (SNR > −9 dB, −6 dB, −3 dB), we observed insignificant performance differences: Se(VF) = 92.5–96.3%, Sp(OR) = 93.4–95.5%, Sp(Asystole) = 92.6–94.0%, *p* > 0.05. Performance stability with respect to CC rate was validated. Generalizable application of the optimized computationally efficient CNN model was justified by an independent OHCA database, which to our knowledge is the largest test dataset with real-life cardiac arrest rhythms during CPR.

## 1. Introduction

The vital steps in the Chain of Survival for managing out-of-hospital cardiac arrests (OHCA) are the prompt provision of high-quality cardiopulmonary resuscitation (CPR), concurrent early defibrillation of ventricular fibrillation (VF) and pulseless ventricular tachycardia [1,2]. High-quality CPR performance includes minimal interruptions of chest compressions (CC) with CC fraction of at least 60%, considering the evidence for improved resuscitation outcomes in patients with shockable rhythms [3,4] and increased return of spontaneous circulation (ROSC) with shorter perishock pauses [5,6]. Conversely, CPR can provide only part of the coronary and cerebral blood flow, and thus the long time spent in VF is potentially lethal [7,8,9]. Therefore, manufacturers of automated external defibrillators (AED) have recently introduced different technologies for efficient management of the resuscitation protocol focused on fewer interruptions of CC, early stopping of CC for VF treatment and/or minimizing the pre-shock pauses [10,11,12,13]. Regardless of the the implementation scheme for shock delivery, stopping, starting or continuing CC, all AED algorithms follow a two-step rhythm detection process, including rhythm analysis during CC and hands-off reconfirmation analysis in absence of CC required in 30–100% of all cases. Thus far, confident shock delivery decisions meeting the American Heart Association’s (AHA) performance goals for AEDs [14] cannot be taken solely during CC due to the strong artifacts that usually disturb the visual and automated diagnostic interpretation of the electrocardiogram (ECG).

The most common strategy for ECG rhythm analysis during CPR is the suppression of CC artifacts in a pre-processor filtering stage based on sophisticated adaptive algorithms (Kalman, least mean squares, recursive least squares). Many adaptive solutions rely on additional reference channels synchronously modulated by the mechanical compressions during CPR, such as:

- impedance or compression depth signal (from an accelerometer) giving a feedback on the instantaneous CC frequency [15,16,17,18,19,20,21,22,23];
- compression force signal [24];
- compression acceleration signal providing information on the CC velocity [25];
- lagged copies of arterial blood pressure signal [26];
- multichannel fusion of compression depth, compression acceleration and thoracic impedance by a recursive adaptive matching pursuit technique [27,28].

Some studies do not use additional sensors, but try to derive the reference input information by estimating the fundamental CC frequency and specific CC components/harmonics in the Fourier power spectral density of the ECG signal itself [29,30]. CC filtering techniques with independent component analysis of over eight ECG leads in animal models has also been shown to be effective [31], although multi-lead configurations are unfeasible for the traditional AED connection to patients in OHCA. Many of the above-referenced studies use artificial mixtures of CC artifacts and ECG recordings under fixed signal-to-noise corruption levels. Only a few of them use real OHCA recordings during CPR, mostly due to the privacy and unavailability of such public electronic records.

In earlier studies, the filtered ECG signals during CPR have been subjected to analysis by conventional shock advisory algorithms validated for noise-free ECGs [18,20,21,22,24,25,28,29,31]. Recent studies instead apply specific feature extraction and optimization strategies of different machine learning classifiers to better deal with the residual CC artifacts that usually persist in the filtered ECG. Particularly, distinguished slope and frequency features are processed by support vector machines (SVM) [19], wavelet features are subjected to classification with linear and quadratic discriminant analysis [32], random forests [15,16], SVM [16,17], kernel logistic regression, boosting of decision trees, shallow neural networks [16].

The use of additional reference channels in almost all rhythm analysis studies with pre-filtering of the CPR artifacts [33,34] is inapplicable in cases of traditional and easy AED connection to the patient directly via the defi-pads without external feedback devices. In such scenarios, the single lead ECG, and eventually the impedance, are the available information channels for the automated shock advisory decision. Considering that the spectra of CC artifacts overlap the dominant VF and QRS components [20,33,35], it has been demonstrated that residual CC artifacts are still present after filtering with, and without, the use of impedance as a reference channel [21,22,36,37]. We should note the potential unreliability of impedance as a reference because it is often prone to noise, as well as fact that the estimated fundamental frequency and morphology of CC artifacts could not match the one observed in ECG. Therefore, special time–frequency techniques for ECG and CC morphology estimation have been applied to evaluate pattern differences directly in the ECG signals; this is larger in presence of VF or disorganized rhythms and smaller in organized rhythms [38,39,40].

In the last few years, deep neural networks (DNN) have demonstrated high potential as end-to-end solutions for feature extraction and accurate classification of cardiac arrest rhythms, mainly concerning the conditions without artifacts [41,42,43,44,45,46]. The most common architectures make use of fully convolutional neural networks (CNN) due to their relatively simple computational profile. Hybrid architectures, including recurrent long short-term memory (LSTM) layers have also been applied, although LSTM is computationally expensive and alone has not demonstrated better performance than fully CNN [38]. In conditions of CPR artifacts, only three recent publications were found to investigate DNNs for detection of shockable (Sh) and non-shockable (NSh) rhythms [47,48,49]. Similarly to their previous studies [15,16,50], Isasi et al. [47,48] rely on the strategy for pre-filtering of CC artifacts by a recursive least squares filter using information for instantaneous CC periods derived either through compression depth signal from external accelerometer sensor or strictly controlled by a mechanical chest compression device. The filtered ECG samples are fed to a CNN model with three convolutional blocks and two fully connected layers for binary classification of Sh/NSh rhythms. In such analysis schemes with pre-filtering, the feature extraction and classification conditions are very similar to analysis of clean high-quality ECGs. Therefore, the presented CNN architectures are not optimized to directly process CC artifacts. The latter important optimization is shown in the study of Hajeb-M et al. [49] for a hybrid DNN architecture, including a combination of convolutional layers, residual blocks and bidirectional LSTM layers. The classification of Sh/NSh rhythms is relying on redundant input information from time and frequency domain ECG representations, such as concatenation of raw ECG samples with amplitude and phase coefficients of short-time Fourier transform. Although the machine learning process and the optimization of the number of residual blocks has been shown in a rigorous manner using cross-validation and controlled signal-to-noise ratio (SNR), a major limitation of this study concerns the missing test results with real-life ECG rhythms and Asystoles (>50% of all interventions) during CPR in OHCA. Such generalization of the trained DNN model is essential, especially in the specific training conditions of [49] applying artificial mix of clean Holter ECG data and CPR artifacts from a limited number of subjects.

The common problems in this subject area cover limited accuracy of all shock advisory algorithms during CPR, especially those related to the simple AED use with a single lead ECG input. Furthermore, the lack of public OHCA databases forces the development and test of algorithms on either proprietary or artificial databases linked to limited CPR sources, artifact morphologies, cardiac arrest rhythms (e.g., lack of Asystoles, although predominant in OHCA; lack of rare Sh rhythms such as ventricular tachycardias) and in overall small database size. All these are obstacles for training of the novel DNN technologies, which are currently the most powerful signal processing tool for feature extraction and classification.

The objective of this study is to optimize the architecture of a computationally efficient end-to-end CNN models for shock advisory decision during CPR using real-life AED recordings in OHCA. The application simplicity is pursued as a minimal input from a single ECG lead acquired directly through the defi-pads without pre-filtering and without additional sensors, and secondly as a simple feature extraction and classification process based on fully convolutional operations. Rigorous random search training and validation of CNN hyperparameters on numerous OHCA interventions aims to select the best performing CNN architecture. Its ability to effectively extract features from raw ECG signals during CPR is comprehensively demonstrated and the dependency on the CPR corruption level in ECG is tested. The results from this study aim to bring light to the generalizable application of the trained CNN model by reporting its performance on a fully independent test database, which to our knowledge is the largest one with real-life cardiac arrest rhythms during CPR.

## 2. Materials and Methods

### 2.1. ECG Databases

**Figure 1:**
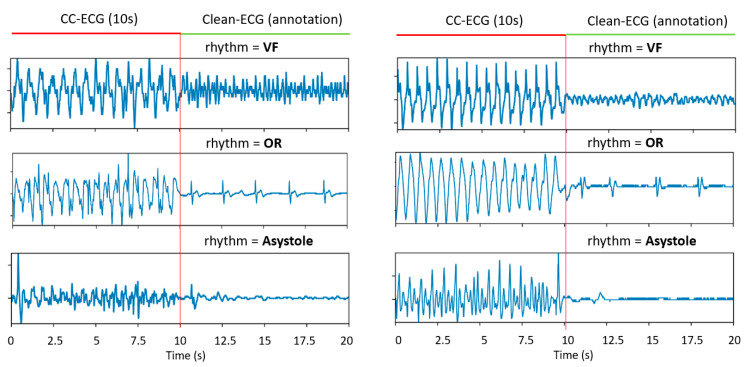
Rhythm annotation scheme during CPR: AED analysis periods (10 s of clean-ECG) were used for rhythm annotation. The rhythm during the preceding period with chest compressions (10 s of CC-ECG) was considered consistent.

The data in this study were retrospectively extracted from ECG electronic records of OHCA interventions with AEDs (FredEasy and Defigard Touch 7, Schiller Médical SAS, Wissembourg, France) used by two French firefighter centers in the cities of Nancy (2005–2010) and Paris (2010–2017). Chest compressions were provided with 30:2 compression-to-ventilation ratio and rate of 100–120 min^−1^, paused every 2 min to run the regular AED rhythm analysis for shock advice, according to the European Research Council (ERC) Adult Basic Life Support guidelines [51]. The ECG signals were acquired in a single lead through the defibrillation pads in antero-apical position on the patient thorax. The rhythm annotation scheme identified the AED analysis periods during the OHCA intervention and used the underlying 10 s of clean-ECG for rhythm observation and annotation by three independent reviewers (Figure 1). After agreement, the annotation of the clean-ECG was accepted for the preceding 10 s period with chest compressions (CC-ECG). This is a common annotation scheme of CC-ECG signals due to presumably unreliable rhythm identification process in conditions of large CC artifacts [11,12,23,34,38,39,40].

The following rhythm categories were considered in this study (Figure 1):

- Coarse ventricular fibrillation (VF) with peak-to-peak ECG amplitude >200 μV;
- Organized rhythm (OR), including normal sinus rhythm, atrial flutter/fibrillation, premature atrial and ventricular contractions, heart blocks, supraventricular tachycardia, sinus bradycardia and idioventricular rhythms;
- Asystole with peak-to-peak ECG amplitude <100 μV.

The data were split patient-wise in independent datasets for training, validation and test with a number of CC-ECG strips with shockable (VF) and non-shockable rhythms (OR and Asystole) as defined in Table 1. The distribution of the rhythms within and between datasets was not controlled but was determined from the content of the OHCA interventions recorded in non-overlapping periods and predefined before the study. The CC-ECG strips were extracted from realistic OHCA scenarios where CPR was provided by different rescuers in:

- 579 interventions between 2005–2010 [38,52] used for training;
- 596 interventions in 2011 [39] used for validation;
- 1545 interventions in 2017 used for the test. These interventions respected a strict inclusion criterion of CC-ECG strips with CC duration ≥10 s before the annotation window. This inclusion criterion was applied in order to guarantee fair report of the test performance of a DNN algorithm that should be run in presence of CC. The presented test database was novel and not used in any previous study.

Further, the CC-ECG signals were explored in the AED monitoring bandwidth (1–30 Hz) without pre-filtering and after down-sampling from 500 Hz to 125 Hz.

For each CC-ECG strip, the corruption level of CC artifacts was estimated relative to the clean-ECG components in the annotation window using the standard metric signal-to-noise ratio (SNR):

(1) $$\mathrm{SNR}=10\log_{10}\frac{\mathrm{power}\;\left(\mathrm{clean}-\mathrm{ECG}\right)}{\mathrm{power}\;\left(\mathrm{CC}-\mathrm{ECG}\right)}\;,$$

where the power represented the variance of the signal with respect to its mean value over 10 s strip duration.

Table 2 presents the estimated content of the test database distributed in four SNR levels, standing for very strong (≤−9 dB), strong (−9 to −6 dB], moderate (−6 to −3 dB] and weak (>−3 dB) CPR artifacts in ECG.

### 2.2. CNN Architecture

The input feature space was a one-dimensional (1D) data vector, representing the one-lead raw CC-ECG signal with duration of 10 s (size L_0_ = 1250 samples). Furthermore, the feature processing stage consisted of a fully convolutional network in a sequence of *N* convolutional blocks. Each block included a 1D Convolution layer (CONV1D), Max-pooling layer (MAXPOOL) and Dropout layer (DROPOUT) as described below:

- CONV1D: The 1D Convolution layer of the i^th^ convolutional block contained F_i_ filters with kernel size (K_i_). The output of the f^th^ filter (f = 1,2,…F_i_) was computed as:
  (2) $${\mathrm{conv}}_{i}^{f}\left[j\right]=\Psi (\overset{K_{i}-1}{\underset{k=0}{\sum }}w_{\mathrm{ki}}^{f}S_{i}\left[j+k\right]+b_{\mathrm{ki}}^{f}),$$
  where:
  - i = [1, 2,…N] identified the sequential number of the convolutional layer;
  - S_i_ was the input vector of the i^th^ convolutional layer, with size (L_i_);
  - j = [0, 1, … L_i_ − K_i_ + 1] indexed the output feature vector, applying convolutional operation with a valid padding [53];
  - $w_{\mathrm{ki}}$, $b_{\mathrm{ki}}$ denoted the weights and biases of the i^th^ convolution kernel, respectively;
  - Ψ was the applied rectified linear unit activation function ReLU.
- MAXPOOL (pool size = MP): Down-sampled the CONV1D layer output ($conv_{i}^{f}$) with size (L_i_ − K_i_ + 1) × F_i_ by applying maximum operation over non-overlapping segments of the feature vector $conv_{i}^{f}$, thus generating a new feature vector $pool_{i}^{f}$ with MP times smaller width ($\frac{L_{i}-K_{i}+1}{\mathrm{MP}}$ ${\times \;F}_{i}$).
- DROPOUT: This regularization layer with a dropout rate α ∈ [0; 1] was applied to avoid overfitting and improved the generalization during training. It generated an output vector $drop_{i}^{f}$ ($\frac{L_{i}-K_{i}+1}{\mathrm{MP}}$ ${\times \;F}_{i}$) with portion of ‘0’ nodes equal to α. In the test process $drop_{i}^{f}=pool_{i}^{f}$, the input signal for the next convolutional layer was *S_i+_*_1_ = $\;drop_{i}^{f}$.

The sequence of N convolutional blocks with the structure described above was followed by Global max-pooling (GMP) layer, which down-sampled $drop_{N}^{f}$ of each filter (f = 1, 2,…F_N_) to a single value equal to its maximal value. Thus, the GMP layer had an output feature size (1 × F_N_), which was fed into a binary classifier implemented as a dense layer (DENSE) with one node, F_N_ weights (w_i_), one bias (b) and a ‘sigmoid’ activation function. The output of the DENSE layer provided the diagnostic probability for presence of shockable rhythm pSh ∈ [0; 1]:

(3) $$\mathrm{pSh}\left(x\right)=\frac{1}{1+e^{-x}},\;\mathrm{where}\;x=\sum_{i=1}^{\mathrm{FN}}w_{i}{\mathrm{GMP}}_{i}+b$$

The interpretation of pSh stands for detected Sh rhythm in case of pSh ≥ pTHR, or detected NSh rhythm otherwise, where pTHR denoted the probability threshold as defined during training (Section 3.2).

The trainable parameters in the proposed DNN architecture could be linked to the weights and biases of the N-blocks CONV1D layers and the final dense layer, and therefore, this model was considered as a fully CNN. The number of trainable parameters could be calculated with the following equation:

(4) $$\mathrm{Params}=\overset{N}{\underset{i=1}{\sum }}F_{i}(K_{i}F_{i-1}+1)+(F_{N}+1).$$

### 2.3. CNN Optimization

**Figure 2:**
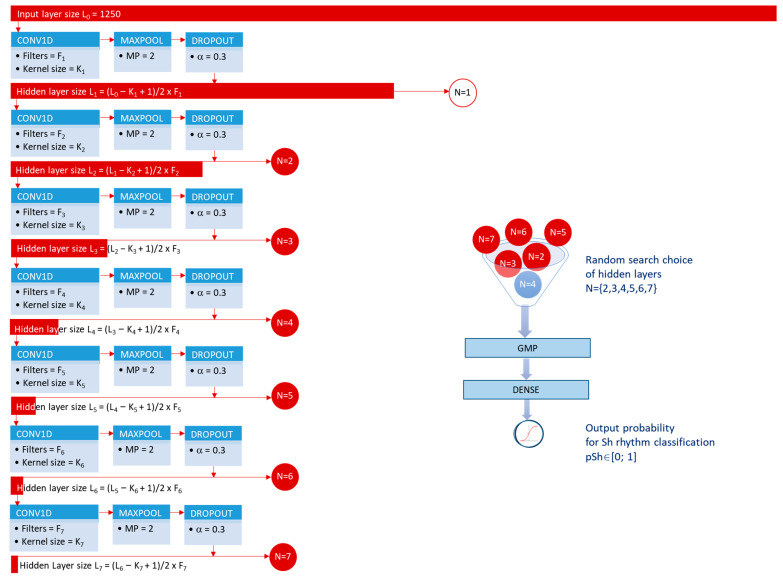
CNN architecture and regularization of the depth by random search selection of the hidden layer output of an arbitrary convolutional block N = {2, 3…7} for classification. The width of the red horizontal bars is scaled to represent the sizes of the input and hidden layers, illustrating the true model shrinkage from top to bottom due to MP = 2 after each convolutional block.

We hypothesized that CNN depth and size influenced the extracted feature map representations of the ECG signal and could have major impact on the classification performance, as well as on the *Params* value and training ability. Therefore, the optimization process considered a flexible CNN architecture with adjustable depth and size, as presented in Figure 2. The CNN depth was regularized by supplying the hidden layer output of an arbitrary convolutional block N directly to GMP and DENSE layer for classification. The CNN size was regularized by setting different number of filters (F_i_) and their kernel sizes (K_i_) at each sequential block (i = 1, 2, …N).

**Figure 3:**
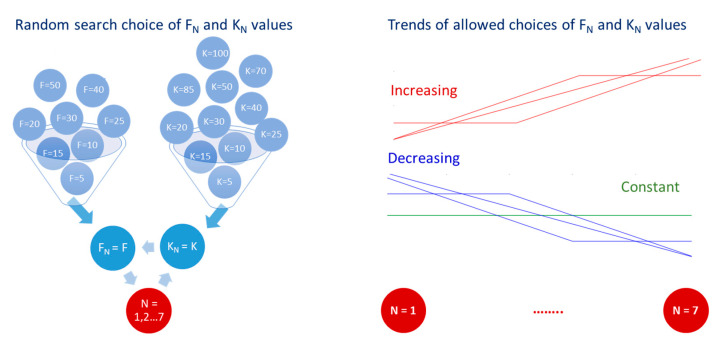
Random search choice of the hyperparameters $\mathrm{HP}={\left\{N,F_{i},K_{i}\right\}}_{i=1}^{N}\;$: grid of values (**left**) and allowed trends of change from first to last CNN layer (**right**). The lines on the right correspond to the seven possible scenarios for driving F_N_ and K_N_ values over layers, i.e., constant (green), continuous increasing (red), constant+continuous increasing (red), continuous+constant increasing (red), continuous decreasing (blue), constant+continuous decreasing (blue), continuous+constant decreasing (blue).

Figure 3 illustrates the defined random search optimization of the hyperparameters $\mathrm{HP}={\left\{N,F_{i},K_{i}\right\}}_{i=1}^{N}\;$ in a large grid of values:

- N = {2, 3, 4, 5, 6, 7};
- F_i_ = {5, 10, 15, 20, 25, 30, 40, 50};
- K_i_ = {5, 10, 15, 20, 25, 30, 40, 50, 60, 70, 85, 100};
- The vectors {F_i_} and {K_i_} were designed to follow a decreasing, increasing or constant trend from top to bottom (i = 1, 2, …N) in the same model (Figure 3).

Figure 2 illustrates the adopted fixed settings in the CNN architecture, including:

- MP = 2 in MAXPOOL layer. This is the minimal value that allowed conditions to build deeper networks by gradual subsampling by two of the feature space after each convolutional block N;
- α = 0.3 in DROPOUT layer. This is the most common dropout setting [53], based also on reports that values of α > 0.3 rapidly increase the error rate [41].

We note that the CNN depth was limited to N = 7 convolutional blocks due to reaching the maximal reasonable model shrinkage after 7 convolutional operations with valid padding and MP = 2. An illustration of the feature size shrinkage from top to bottom of the model is shown in Figure 2 (horizontal red bars). Considering the input feature vector of 1250 samples and minimal kernel size K = 5 at all CONV1D layers, the feature vector after the seventh convolutional block would have maximal size of 5 samples. This value was considered insufficient for optimization of subsequent convolutional blocks.

In the process of random search HP optimization, several models were trained under equal conditions (Section 2.3) on the training dataset. Further, they were ranked according to their performance on the validation dataset. Standard performance metrics for reporting detection accuracy of Sh and NSh rhythms in AEDs [14] were computed, including sensitivity (Se) and specificity (Sp):

(5) $$\mathrm{Se}=\frac{\mathrm{TP}}{\mathrm{TP}+\mathrm{FN}},\;\mathrm{Sp}=\frac{\mathrm{TN}}{\mathrm{TN}+\mathrm{FP}}\;,$$

where True Positives (TP) are the correctly detected Sh cases; False Negatives (FN) are the Sh cases classified as NSh; True Negatives (TN) are the correctly detected NSh cases; False Positives (FP) are the NSh cases classified as Sh.

The best performing random search model was selected by the formal rules for reporting the highest pair (Se, Sp) on the receiver operating characteristic (ROC) curve, namely maximal balanced accuracy (BAC):

(6) $$\mathrm{HPbest}:\;\mathrm{BAC}=\frac{\mathrm{Se}+\mathrm{Sp}}{2}\to \max$$

### 2.4. CNN Training

CNN models design, training and evaluation was embedded in TensorFlow framework using Keras built-in APIs. The training was done with balanced dataset obtained by over-sampling of the minority class, i.e., replicating six times the number of Sh cases related to the ratio of 2593 NSh to 408 Sh cases in Table 1. The dataset was randomized by shuffling and was fed into batches with size 256. Normalization of the input data was not applied and the input signal resolution of 5 μV/LSB was maintained. This was purposely done in order to keep the real ECG amplitude since it is characteristic for some of the analyzed rhythms (e.g., Asystole peak-to-peak amplitude < 100 µV). Each model was trained in at most 400 epochs, considering that early stopping was applied if no improvement in performance was observed for more than 150 epochs. For the model design and fitting, we applied: ‘Random uniform’ kernel initializer, ‘Adam’ optimizer with learning rate of 0.001, exponential decay rate for the first and second moment estimates β1 = 0.9 and β2 = 0.999, respectively. The loss function was ‘Binary cross-entropy’ for 2 target classes (Sh/NSh) and the accuracy metrics function corresponded to BAC. For each setting of $\mathrm{HP}={\left\{N,F_{i},K_{i}\right\}}_{i=1}^{N}\;$, the model with maximal accuracy after all training epochs was stored in HDF5 file. All experiments were conducted in a workstation with Intel CPU Xeon Silver 4214R @ 2.4 GHz (2 processors), 96 GB RAM, NVIDIA Quadro K4000-3Gb GPU.

## 3. Results

### 3.1. HP Optimization

The optimization process was run to generate a total number of 1500 CNN models with a random search of $\mathrm{HP}={\left\{N,F_{i},K_{i}\right\}}_{i=1}^{N}$ within the defined grid. The distributions of the number of trained CNN models and the training epochs for their best performance on the validation set is summarized in Table 3. We observed earlier training for deeper models, justified by the gradual trend for reducing the number of training epochs, starting from N = 3 to 7 convolutional blocks. Deduced from the upper quartile, the maximal number of 400 training epochs could be considered sufficient for training.

**Figure 4:**
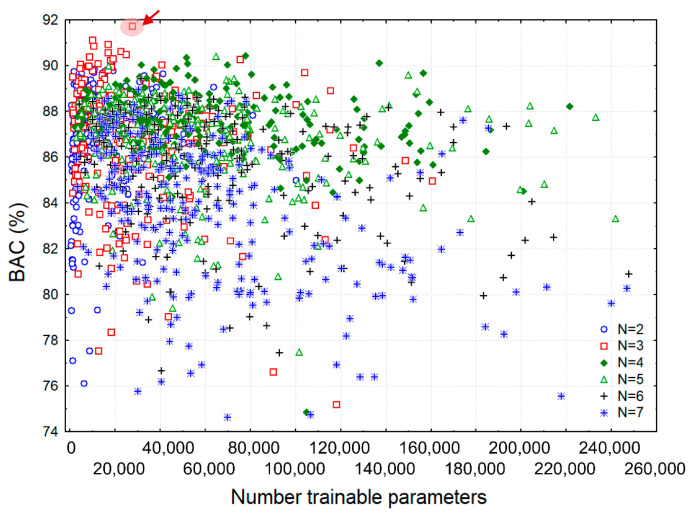
Validation BAC performance of all random search CNN models in function of the number of trainable parameters. The scatter plot is depicted in six groups for the number of convolutional blocks N = {2, 3, 4, 5, 6, 7}. The highlighted scatter point is corresponding to the best performance model CNN3-CC-ECG.

During training, the number of trainable parameters (Params in Equation (4)) was limited to a maximum of 250,000 in order to reduce the computational cost as it was found that redundant models did not produce high accuracy. This could be deduced from Figure 4, which is a representation of the validation performances of all generated random search models in function of their trainable parameters. We observed a dense overlap of good models with similar performances of BAC = 86–89% at limited Params < 80,000, although the models had different depths from N = 2 to 7 convolutional blocks. The best models with BAC = 90–92% were, however, mostly observed for N = 3 convolutional blocks, and could be located in the optimal zone of low-complexity models with Params < 30,000 (the top-left corner of Figure 4).

**Figure 5:**
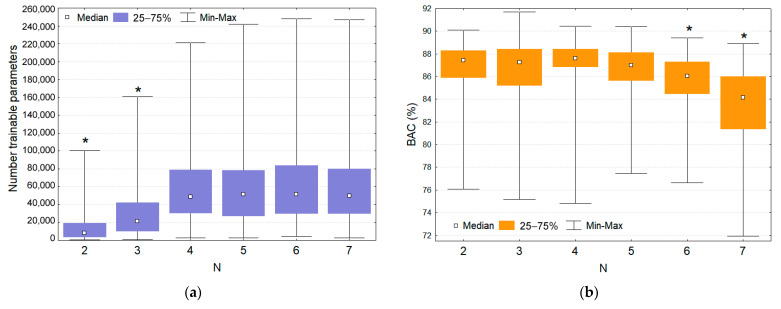
Categorical analysis of random search models: (**a**) Number of trainable parameters; (**b**) Validation BAC performance, according to the number of convolutional blocks N = {2, 3, 4, 5, 6, 7}. The statistically different distributions are highlighted at *p* < 0.05 (*).

The influence of the number of convolutional blocks on both the model complexity in terms of trainable parameters (Figure 5a) and the chosen performance measure on the validation dataset BAC (Figure 5b) was statistically evaluated with one-way analysis of variance (ANOVA). The Bonferroni post-hoc test justified the benefit of the shallowest models with 2 and 3 convolutional blocks, which presented significantly lower number of trainable parameters (*p* < 0.05) while keeping the highest level of performance. The latter was observed for all models with 2 to 5 convolutional blocks, which presented the top BAC distributions with insignificant differences (*p* > 0.05). The deepest models with 6 and 7 convolutional blocks were, however, associated with significant drop of performance (*p* < 0.05), although they had similar complexity as those with 4 and 5 convolutional blocks. The above observations suggested that maximal shrinkage of the feature space in deepest models had deteriorating impact on performance.

### 3.2. Optimal CNN Model

Considering the optimal zone of BAC→max in Figure 4, we selected one of the models with three convolutional layers as the final optimal CNN model for ECG analysis during CPR chest compressions, further named CNN3-CC-ECG network. Description of its hyperparameters, number of trainable parameters and output shape is presented in Table 4.

**Figure 6:**
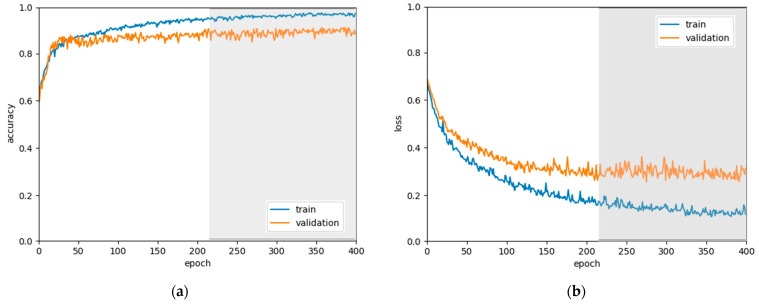
Training process of CNN3-CC-ECG network run over 400 epochs, showing the accuracy (**a**) and loss (**b**) curves for the training and validation dataset. The best model (validation accuracy→max) was achieved in 213 epochs. Further training epochs were discarded as being ineffective (gray area).

Figure 6 shows the training epochs of CNN3-CC-ECG network with accuracy and loss curves relative to the training and validation databases. The training of the model attained maximal accuracy on the validation database in 213 epochs. Further overfit on the training dataset was not considered as no essential trend for minimizing the validation loss was observed (the gray zone in Figure 6).

**Figure 7:**
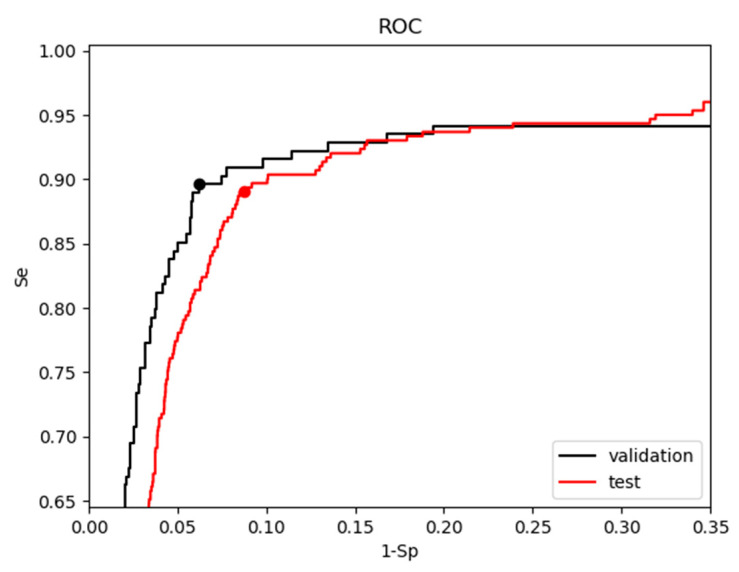
ROC curves of CNN3-CC-ECG network for the validation and test databases. The threshold of the ROC operating point for the validation database (black ‘o’ mark) was computed at BAC→max. The same threshold was applied to report the final performance for the test database (red ‘o’ mark).

Figure 7 illustrates the ROC curves of CNN3-CC-ECG network on the validation and test databases with Area Under the Curve ROC-AUC equal to 0.945 and 0.938, respectively. The condition (BAC→max) was applied to the validation ROC to define the probability threshold (pTHR = 0.74) of the operating point at which the final performance on all databases (training, validation and test) was reported (Table 5). The independent evaluation on the test dataset shows relatively small drop in performance compared to the validation database, i.e., Se drop of 0.4% points (89% vs. 89.4%), Sp drop of 2.5% points (91.3% vs. 93.8%). This justifies the generalizability of CNN3-CC-ECG network to take shock advisory decisions during CPR with accuracy BAC = 90.2% deduced from nearly 5600 real OHCA ECG strips.

### 3.3. CNN Features

This section gives insight to the hidden layer features, which have been automatically trained in our optimized network with respect to their merit for discrimination of Sh and NSh rhythms. We note that this interesting feature extraction process in CNNs usually remains uncovered in most of the published deeper architectures, which just apply raw ECG data at the input and observe the output diagnostic probability. Such view is reasonable in our study due to the relatively simple CNN3-CC-ECG architecture, and is further used to show the reasons for some errors.

**Figure 8:**
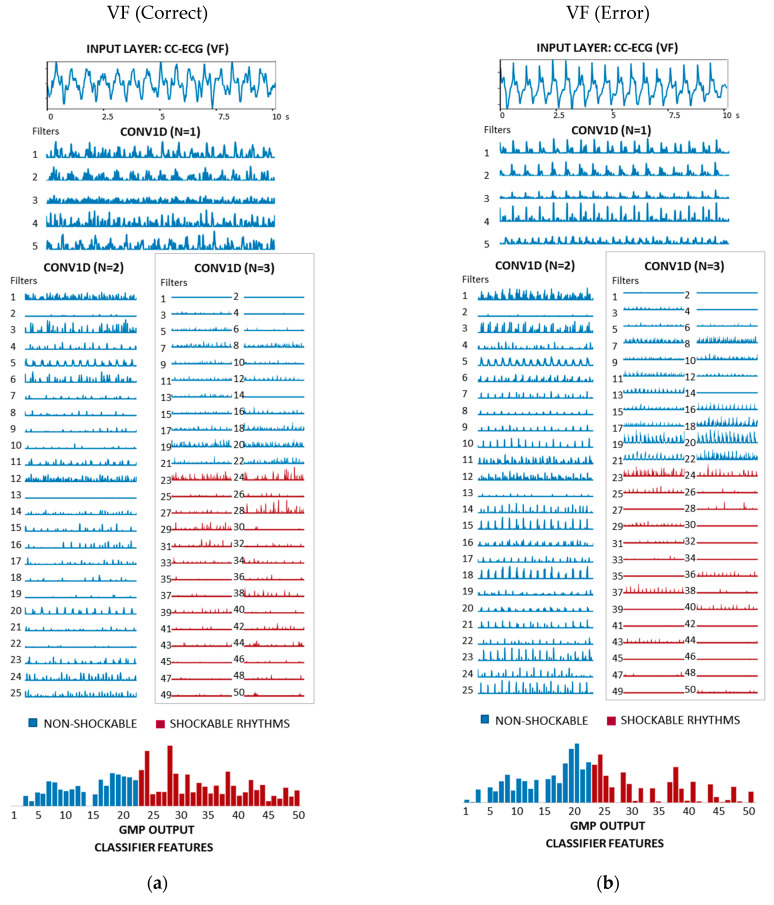
Features of CNN3-CC-ECG network computed for two examples of 10 s ECG-CC strips annotated as VF (Figure 1) with different shock advisory decisions: (**a**) VF (SNR = −6.5 dB) correctly detected as Sh rhythm with output probability pSh = 0.97; (**b**) VF (SNR = −12.9 dB) erroneously detected as NSh rhythm with output probability pSh = 0.007. The underlined 50 filters at the output of CONV1D (N = 3) are used for computation of 50 classifier features (bottom plot), grouped for Sh (red) and NSh (blue) rhythms in accordance with the sign of the classifier weights.

**Figure 9:**
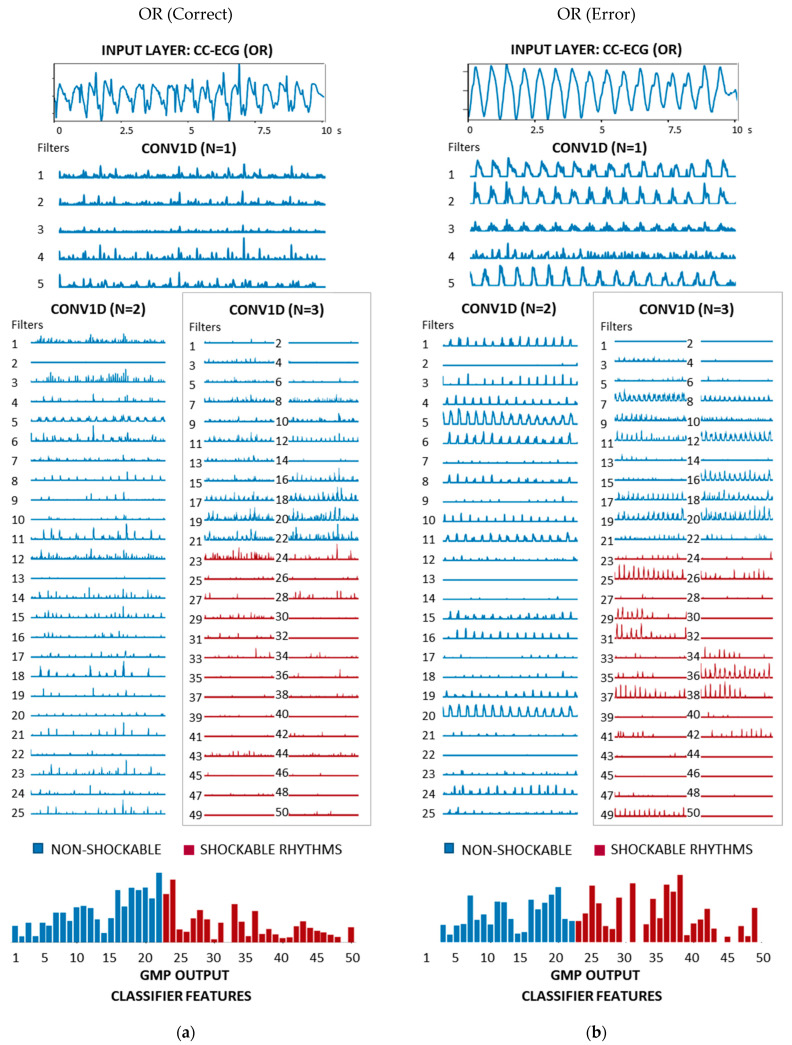
Features of CNN3-CC-ECG network computed for two examples of 10 s ECG-CC strips annotated as OR (Figure 1) with different shock advisory decisions: (**a**) OR (SNR = −14.6 dB) correctly detected as NSh rhythm with output probability pSh = 0.001; (**b**) OR (SNR = −18.4 dB) erroneously detected as Sh rhythm with output probability pSh = 0.858. The underlined 50 filters at the output of CONV1D (N = 3) are used for computation of 50 classifier features (bottom plot), grouped for Sh (red) and NSh (blue) rhythms in accordance with the sign of the classifier weights.

**Figure 10:**
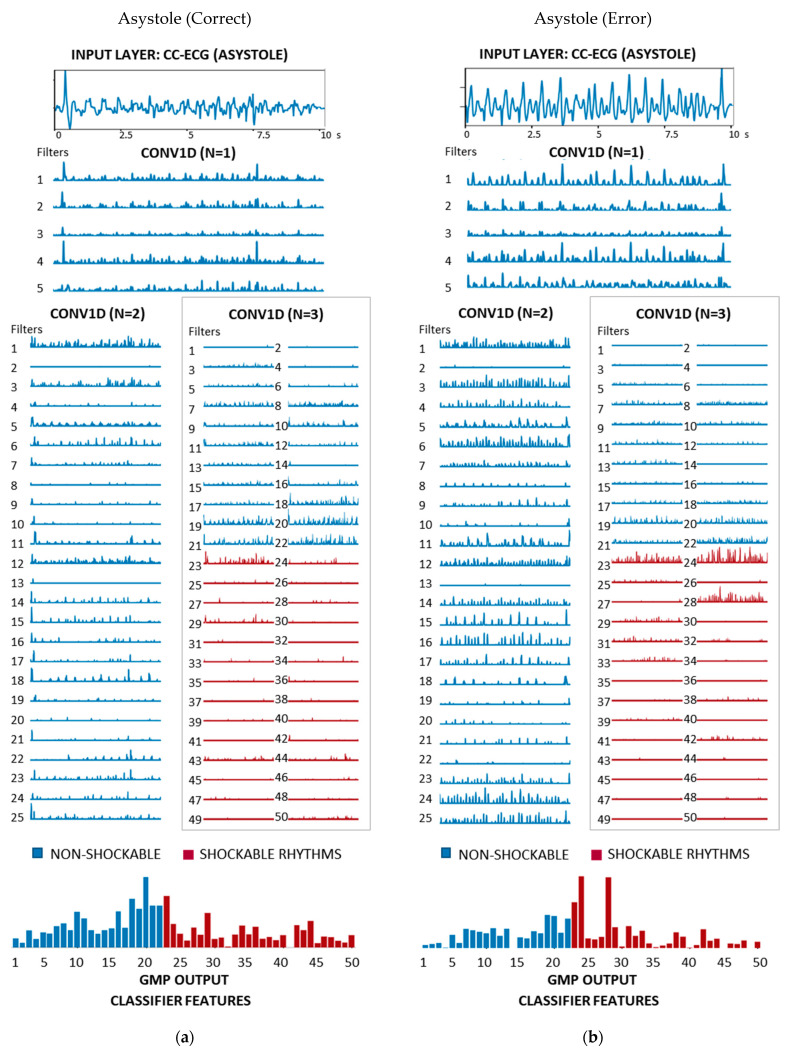
Features of CNN3-CC-ECG network computed for two examples of 10 s ECG-CC strips annotated as Asystole (Figure 1) with different shock advisory decisions: (**a**) Asystole (SNR = −18 dB) correctly detected as NSh rhythm with output probability pSh = 0.006; (**b**) Asystole (SNR = −24 dB) erroneously detected as Sh rhythm with output probability pSh = 0.81. The underlined 50 filters at the output of CONV1D (N = 3) are used for computation of 50 classifier features (bottom plot), grouped for Sh (red) and NSh (blue) rhythms in accordance to the sign of the classifier weights.

Figure 8, Figure 9 and Figure 10 show a comprehensive illustration of the features extracted in the hidden layers of the optimized CNN3-CC-ECG network for all rhythms in Figure 1. Intermediate features are observed in the output of the three CONV1D layers: 5 filters with 1241 samples (N = 1), 25 filters with 601 samples (N = 2), and 50 filters with 281 samples (N = 3). They could be interpreted in terms of non-linear filtering after convolutional operation and ReLU activation, giving responses in specific frequency bands adjusted by training of the network. Although the filtering of CC artifacts was not the goal in the process of CNN3-CC-ECG training, it would be interesting to observe how convolutional layers succeed to filter out CC artifact components and expand ECG components. Convolutional filter outputs could be unambiguously interpreted after the third CONV1D layer (N = 3) with 50 filters, because they are directly used by GMP to derive the 50 final features input to the classifier. For better interpretation of Figure 8, Figure 9 and Figure 10, the 50 filters in CONV1D layer (N = 3) and the derived from them 50 GMP features were ordered in respect to the classifier weights (Equation (3)), starting from the most negative to the most positive weight. According to Equation (3), negative classifier weights lead to decreasing of pSh and thus contribute to NSh output; while positive weights lead to increasing of pSh and thus contribute to Sh output. Particularly in CNN3-CC-ECG model, filters and GMP features numbered from 1 to 22 are associated with negative classifier weights and high GMP values are beneficial for the detection of OR and Asystole signals. Alternatively, filters and GMP features numbered from 23 to 50 are associated with positive classifier weights and high GMP values are beneficial for the detection of VF signals.

Figure 8 illustrates two VF examples, representative for a true positive and a false negative detection with estimated output probability pSh = 0.97 and pSh = 0.007, respectively. The correctly detected VF (Figure 8a) has visible VF components during CC (SNR = −6.5 dB) and CONV1D filters succeed to present them in different modalities so that Sh features dominate over NSh features (bottom plot). The CC artifacts in the erroneously detected VF (Figure 8b) have a very sharp uncommon morphology, which resembles a periodic QRS behavior at the output of CONV1D (N = 1). These morphologies lead to high response at the output of 16–22 CONV1D filters (N = 3) and high values of the respective NSh features. Unfavorably, the VF amplitude is low (SNR = −12.9 dB) and leads to low estimation of many Sh features.

Figure 9 illustrates two OR examples, representing a true negative and a false positive case with pSh = 0.001 and pSh = 0.858, respectively. Although the SNR is low (SNR = −14.6 dB), the correctly detected OR (Figure 9a) has visible QRS components during CC and CONV1D filters succeed to present them in different modalities so that NSh features dominate over Sh features (bottom plot). The CPR artifact of the erroneously detected OR (Figure 9b) resembles a slow dominant sinusoidal wave without visible QRS complexes (SNR = −18.4 dB). We observe that many filters with expected high output for Sh rhythms are activated so that Sh features become more dominant than NSh features.

Figure 10 illustrates a true negative and a false positive example of Asystole with low SNR < −18 dB and output probability pSh = 0.006 and pSh = 0.81, respectively. Although the CC artifacts in the correctly detected Asystole (Figure 10a) have a morphology, which could misleadingly interpreted as VF in parts, the filters succeed to suppress those components so that the Sh features receive low estimation. The CPR components of the erroneously detected Asystole (Figure 10b) have a morphology, which also resembles VF, but it appears enhanced with the very high-amplitude output in three Sh filters (numbered 23, 24, 28). This leads to a higher prevalence of Sh than NSh features, and the estimated probability for presence of shockable rhythm *pSh* = 0.81 appears slightly above the defined threshold pTHR *=* 0.74.

### 3.4. SNR Impact

This section investigates the impact of the CPR artifact corruption level on the CNN3-CC-ECG network performance (features and accuracy) using the test set.

At the baseline, the CPR corruption level is measured for different rhythms in the test set, further reported as SNR mean value ± standard deviation (min-max range): VF (−6.7 ± 4.8 dB, −19.7 to 4.6 dB), OR (−6.3 ± 6.4 dB, −31 to 15 dB), Asystole (−22.4 ± 10.1 dB, −60 to 25 dB). Bonferroni post-hoc tests in ANOVA do not find substantial differences of the SNR in both groups with heart electrical activity (either organized in OR, or not organized in VF), *p* > 0.05. The CPR corruption level is found significantly higher only in the group of Asystoles, with amplitudes of the ECG components <100 μV (*p* < 0.0001).

**Figure 11:**
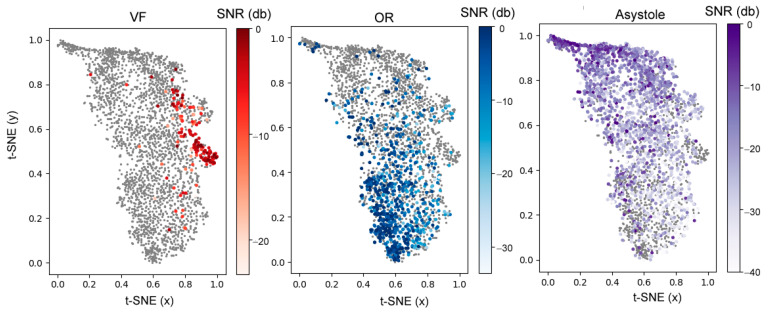
2D t-SNE distribution of GMP features in CNN3-CC-ECG network, estimated for the test database for different rhythms (OR, Asystole, VF). The color gradients represent the distribution of the features with respect to SNR.

In the second step, we investigate the dependency of the extracted network features (GMP output input to the classifier) on SNR. For a more comprehensive view of 50 GMP features, they are reduced to a two-dimensional (2D) array by means of the t-distributed stochastic neighbor embedding algorithm (t-SNE) [54]. As illustrated in Figure 11, the 2D feature array exhibits separable clusters for rhythms VF, OR and Asystole, although they present a certain level of overlap. As a potential source of errors, the overlap between clusters (VF, Asystole) or (VF, OR) could be better interpreted when SNR is accounted. The color gradient from dark (high SNR) to light (low SNR) distinguishes well-separable clusters with high SNR at the top (Asystole), at the bottom (OR) or center-right (VF). We observe a potentially problematic area with notable overlap between VF and NSh rhythms with low SNR (t-SNE x > 0.7, 0.8 > y > 0.55).

**Figure 12:**
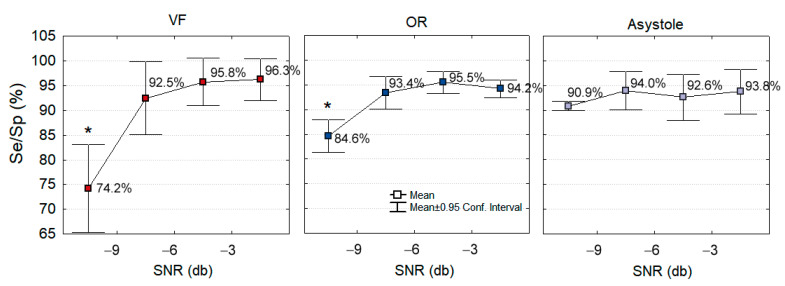
Dependency of CNN3-CC-ECG network performance on the CPR artifact corruption level in ECG estimated on the test database. The mean value and 95% confidence interval of Se (VF) and Sp (OR, Asystole) are presented in function of four SNR levels. Significant drop of performance is highlighted for Se (VF) and Sp (OR) at SNR < −9 dB (* *p* < 0.05).

Finally, we investigate the influence of SNR on the CNN3-CC-ECG network accuracy. In Figure 12, the mean value and 95% confidence intervals of Se (VF) and Sp (OR, Asystole) are presented in function of four SNR levels, standing for very strong (≤−9 dB), strong (−9 to −6 dB], moderate (−6 to −3 dB] and weak (>−3 dB) CPR artifacts in ECG as computed for the test set samples in Table 2. In general, the Sh/NSh rhythm detection is maximal in weak CPR artifacts with mean Se about 96% and mean Sp about 94%. While the CPR artifact corruption level is increasing, the performance is gradually decreasing. This decrease is insignificant for Asystole at all SNR (Sp = 90.9% vs. 94%, *p* > 0.05), as well as for VF and OR at SNR > −9 dB (Se = 92.5% vs. 96.3%, Sp = 93.4% vs. 95.5%, *p* > 0.05). We denote significant drop of Se = 74.2% (VF) and Sp = 84.6% (OR) at very strong CPR artifacts with SNR ≤ −9 dB, *p* < 0.05.

### 3.5. Impact of the Chest Compression Rate

This section investigates the influence of the CC rate on the CNN3-CC-ECG network performance on the test set. Expectedly, CC rate is similar for different rhythms (mean ± standard deviation): 110.3 ± 13.5 min^−1^ (VF), 111.4 ± 16.9 min^−1^ (OR), 111.3 ± 19.0 min^−1^ (Asystole), *p* > 0.05. The mean values closely correspond to the metronome rate of 110 min^−1^, which should be normally followed by rescuers during the resuscitation protocol.

**Figure 13:**
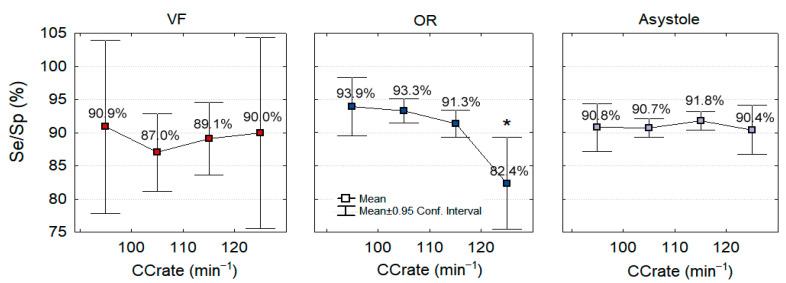
Dependency of CNN3-CC-ECG network performance on the CC rate in the test database. The mean value and 95% confidence interval of Se (VF) and Sp (OR, Asystole) are presented in function of four CC rate ranges: slow (<100 min^−1^), normal (100–110 min^−1^, 110–120 min^−1^) and rapid compressions (>120 min^−1^). Significant drop of performance is highlighted for Sp (OR) at CC rate >120 min^−1^ (* *p* < 0.05).

Figure 13 presents the mean value and 95% confidence intervals of Se (VF) and Sp (OR, Asystole) in the function of CC rate, covering slow (<100 min^−1^), normal (100–110 min^−1^, 110–120 min^−1^) and rapid compressions (>120 min^−1^). We denote a significant drop of Sp = 82.4% (OR) only at rapid compressions >120 min^−1^, *p* < 0.05. Otherwise, all other rhythms and CC rates present insignificant variation of Se = 87–90.9% (VF), Sp = 90.4–91.8% (Asystole), as well as Sp = 91.3–93.9% (OR) for CC rates <120 min^−1^, *p* > 0.05. These results give confidence that performance stability for different CC rates could reassure generalizability despite the known overlap of CPR and ECG rhythm components.

## 4. Discussion

This study provides evidence for the optimization of a deep learning technology in the task of shock advisory analysis of cardiac arrest rhythms (VF, OR and Asystole) during continuous CC. We investigate the potential of a standard convolutional architecture with optimized HPs (depth, filters and kernels at each layer) to self-extract meaningful ECG components in conditions of real-life CC artifacts. Detection of Sh and NSh arrhythmia in such noisy conditions is traditionally difficult because CC artifacts corrupt the ECG channel to such an extent that ECG rhythms frequently appear unrecognized even by medical experts, as illustrated in the examples of Figure 1.

To our knowledge, this is the first study which shows rigorous training, validation and test of CNN models with large independent databases from realistic OHCA scenarios during CPR, taking only the single lead ECG through defi-pads. Such database setting is indispensable in deep learning, which requires a vast amount of training, validation and test data to be effective and to justify generalizable results. The limited number of studies in this area is mostly due to publicly unavailable data with real-life cardiac arrest rhythms during CPR recorded by AEDs used in different OHCA scenes and rescuers.

We implement a simple end-to-end fully CNN architecture, applying raw CC-ECG signals at the input and obtaining Sh probability at the output, without pre-filtering or additional sensors. After HP random search of 1500 CNN models with up to 7 convolutional layers, up to 50 filters, and up to 100 kernel sizes, the optimization results on the validation set show:

- Significant inferiority of all deepest models with 6 and 7 convolutional layers, suggesting that maximal shrinkage of the feature space has deteriorating impact on performance (Figure 5b).
- Distinguishable superiority of several models with three convolutional layers (Figure 4) among which we select the best performance CNN architecture CNN3-CC-ECG (Table 4).

Our optimization goal is based on maximal BAC, which corresponds to the most convex point of ROC (Se+Sp→max, Figure 7). This optimization score is beneficial for maximizing together both Se and Sp, as only those statistical indices have threshold requirements in the AHA performance goals [14]. Generally, BAC assumes equal proportion of false detections within both classes (Sh and NSh), leading to larger absolute number of FPs in the larger NSh class (706 OR+Asystoles in the validation dataset), which is about five times larger than Sh class (151 VF in the validation dataset), i.e., BAC assumes imbalanced number of FP and FN, proportional to the class size. In contrast, other statistical metrics known to deal with imbalanced classes, such as F1-score = 2TP/(2TP + FP + FN) would assume balanced number of FP and FN at the expense of increasing the number of FNs. This could lead to intolerable Se drop below the AHA goals [14].

We justify the generalizability of CNN3-CC-ECG network by independent test on a large real-life OHCA dataset, reporting Se = 89% (268/301 VF), Sp = 91.7% (1504/1640 OR) and Sp = 91.1% (3325/3650 Asystole) as seen in Table 5. The validation to test performance drop is estimated to be about 1.4% points (BAC), which is important to account for while considering the common question of the stability of DNN performance on databases from different subjects and interventions. At the least, performance stability on databases across years could reassure generalizability despite of susceptible fit to specific AED models. In this context, we justify performance stability with respect to CC rate (Figure 13), which could vary due to objective or subjective circumstances (e.g., change of resuscitation policies across years, different settings of metronome rates in specific AED models, rescuers’ abilities for providing CC with and without metronome guidance).

In Table 6, the performance of CNN3-CC-ECG network is compared to other published methods for detection of Sh and NSh rhythms during CPR. Those reference methods have been selected because they use test set from human ECGs and either have analyzed only ECG signals without the need for additional channels for CPR assessment, or have performed the rhythm classification via neural networks.

Although the notable disparities between the studies in Table 6, concerning different test conditions and databases, their comparison reveals that neural networks (this study and [47,48,49]) present equal or better performance than traditional machine learning classifiers [29,38,40] for analysis of cardiac arrest rhythms during CPR. Formally, the relatively simple fully convolutional architecture of CNN3-CC-ECG network in this study shows similar performance to a hybrid DNN architecture [49] (including convolutional layers, residual blocks and bidirectional LSTM layers), i.e., Se of the hybrid DNN model is 5.2% points higher than CNN3-CC-ECG (94.2% vs. 89%), while Sp of CNN3-CC-ECG is 5.2% points better than the hybrid DNN network (91.3% vs. 86.1%). Considering the denoted dependency of performance on SNR (Figure 12), a fair comparison should count the SNR value in both studies. The hybrid DNN was tested in artificial conditions with fixed SNR = −3 dB, which falls within the group of moderate CC artifacts in Figure 12 (SNR between −6 and −3 dB). In these conditions, CNN3-CC-ECG network reveals better Se of about 1.6% points (95.8% vs. 94.2%) for VF and better Sp of about 9.4% points (95.5% vs. 86.1%) for OR. Asystoles have not been tested in [49], although they are the most common rhythm in cardiac arrests that define the border amplitude between Sh and NSh rhythms, and therefore, are important for adequate training of end-to-end DNN classifiers. The other two studies with neural networks [47,48] present similar architecture as CNN3-CC-ECG with three convolutional layers, although the max-pooling size, number of filters and kernel sizes are different. It is worthy to note that the input to the networks is different, i.e., CNN3-CC-ECG network is optimized to directly process ECG with CC artifacts, while the networks in [47,48] are optimized to process ECG after adaptive filtration with the help of an external sensor. The latter network is linked to a specific signal processing chain, which is hardly reproducible without that external sensor. The total benefit of that analysis chain is undisputable, although the network inputs clean-ECG so that the contribution of the network itself for processing of CC artifacts is obscure. Instead, CNN3-CC-ECG network claims to have a self-sufficient architecture, which is able to optimally extract ECG features during CPR and to independently make a shock advisory decision. It is noteworthy, as we report the final CNN3-CC-ECG network performance on an independent real-life test database, that other deep learning studies disclose results from cross-validation [47,49] or random repetitions [48] using the validation dataset.

Ten seconds analysis duration in this study is within the range used in other studies, varying from 8 s to 14 s (Table 6). We consider it relevant to use more rhythm information from longer input ECG segments rather than shrinking the input by shortening the analysis interval. The latter has been shown to reduce accuracy [47] and is not justified in conditions of continuous CC as soon as the highest possible accuracy is beneficial to avoid unnecessary interruptions of CC. We shrink the input feature space by down-sampling to 125 Hz, which is considered to be the reasonable limit of the sampling rate that will not affect ECG components in cardiac arrest rhythms.

End-to-end neural networks are usually considered as black boxes and the extracted features in their hidden layers are taken almost blindly, without comprehensive explanation, supervised control or expectations for the features’ optimal values. Such an approach is beneficial, firstly because it provides simple management of the raw signal input and direct view to the classifier output performance, without going through the bulky feature extraction and selection process. Secondly, it allows the unsupervised computation of abundance of novel features different than the standard definitions based on medical and engineering interpretations. This plethora of novel features found in hidden layers are, however, rarely illustrated or discussed in studies, even though they are the only tool for a comprehensive view on the reasons for false positive or false negative classified cases at the output layer. The optimized network in our study is relatively simple, and therefore, we are able to make a comprehensive illustration of all features for several examples with correct and incorrect shock advisory decisions (Figure 8, Figure 9 and Figure 10). We turn attention to the self-extracted features after convolutional filters, illustrating their relevance for the enhancement of ECG components specific to the rhythm and for the suppression of the corrupting CC artifacts. The noted reasons for errors are due to the diverse morphology of CC artifacts induced in ECG majorly through the skin-electrode interface [55] pushed arbitrarily by different rescuers. The resultant CC artifact waveform can either resemble periodic QRS components (Figure 8b) or VF wave components (Figure 9b and Figure 10b). These CC artifact components can produce high output of CONV1D filters (N = 3), which are shown to be responsible either for NSh (filter numbers 1–22) or Sh (filter numbers 23–50) feature extraction. In very strong artifacts with small SNR < −9 dB, the intrinsic CC artifact morphology plays a significant role and leads to a drop in accuracy for Se (VF) by 18% points and Sp (OR) by 9% points (Figure 12). Otherwise, for strong, moderate and weak CC artifacts (SNR > −9 dB, −6 dB, −3 dB); the filters’ output response is more adequate to represent true ECG components, which is justified by the insignificant change of CNN3-CC-ECG network performance: Se(VF) = 92.5–96.3% and Sp(OR) = 93.4–95.5%, Sp(Asystole) = 92.6–94.0%. Compared to traditional machine learning [29,38,40], the deep learning technology trained and tested with realistic OHCA signals succeed to improve Sp by about 3–10% points, however, the total performance still does not meet the AHA recommendations for AED shock advisory decisions in not-noisy conditions [14]. In addition, the overlapping feature clusters (Figure 11) suggest that CNN technology using solely input ECG information is not able to completely discriminate VF, OR and Asystole components during CPR and could not provide a reliable enough shock advisory decision alone.

## 5. Limitations

Although our test database was extracted from 1545 OHCA patients, it did not contain statistically valuable number of shockable ventricular tachycardia (11 cases representative to less than 0.2% of the total number of rhythms). Therefore, we were unable to fulfil the minimal sample size of the AHA performance goals [14] for reporting statistically significant Se results for ventricular tachycardia based on this database. This phenomenon could be partially linked to the fixed data collection period (one year) which limits the possibility to extend the collection of some rare rhythms.

## 6. Conclusions

Solving the task for shock advisory rhythm analysis during CPR, this study presented the optimal architecture of a deep CNN with best performance among 1500 random search models with 2 to 7 convolutional layers, 5 to 50 filters and 5–100 kernel sizes based on a rigorous training and optimization on large OHCA databases. Generalizable application of the optimized CNN3-CC-ECG network was justified in an independent OHCA database, which to our knowledge is the largest test dataset with real-life cardiac arrest rhythms during CPR with 5591 CC-ECG samples. Although the deep learning with raw ECG input succeed in improving Sp by about 3–10% points compared to traditional machine learning with hand-crafted features, the total performance is lower than AHA recommendations for AED shock advisory decisions in not-noisy conditions by about 1% points for VF, 3.7% points for OR and 3.9% points for Asystole. It could be suggested that there is a room for performance improvement by training data augmentation or design update, including more sophisticated layers and additional channels. Nevertheless, the presented network was designed to be compatible with the simplest and computationally efficient DNN architecture for real-life AED applications.

## Figures and Tables

**Table 1:** Number of ECG strips with CC artifacts extracted from OHCA interventions in different rhythm categories. Independent interventions from different patients were used in the training, validation and test databases.

	Training Database	Validation Database	Test Database
Shockable rhythms			
VF	408	151	301
Non-shockable rhythms			
OR	1089	706	1640
Asystole	1504	1671	3650

**Table 2:** Number of ECG strips in the test databases counted in four SNR levels.

	−9 dB ≤ SNR	−9 dB < SNR ≤ −6 dB	−6 dB < SNR ≤ −3 dB	SNR > −3 dB
Shockable rhythms				
VF	97	53	71	80
Non-shockable rhythms				
OR	456	226	335	623
Asystole	3266	149	122	113

**Table 3:** Trained CNN models by HP random search.

Number of Convolutional Blocks (N)	Number of Trained Models	Training Epochs Median (Quartile Range)
2	259	204 (131–288)
3	231	216 (137–291)
4	243	186 (113–266)
5	253	148 (83–243)
6	240	131 (86–227)
7	274	104 (58–210)

**Table 4:** Details of CNN3-CC-ECG network for analysis of ECG signals during CPR.

Layer Type		Description	Params	Output Shape
Input		-	-	(1250, 1)
Convolutional blocks	N = 1	K_1_ = 5, F_1_ = 5, ReLU MP = 2, α = 0.3	55	(620, 5)
	N = 2	K_2_ = 20, F_2_ = 25, ReLU MP = 2, α = 0.3	2525	(300, 25)
	N = 3	K_3_ = 20, F_3_ = 50, ReLU MP = 2, α = 0.3	25,050	(140, 50)
GMP		-	-	(50)
DENSE		Units = 1, Sigmoid	51	(1)

**Table 5:** Performance of CNN3-CC-ECG network for analysis of ECG rhythms during CPR.

Performance	Training Database	Validation Database	Test Database
Se (Shockable rhythms: VF)	100% (408/408)	89.4% (135/151)	89.0% (268/301)
Sp (Non-shockable rhythms)	97.1% (2518/2593)	93.8% (2230/2377)	91.3% (4830/5290)
Sp (OR)	97.0% (1056/1089)	94.9% (670/706)	91.7% (1504/1640)
Sp (Asystole)	97.1% (1460/1504)	93.4% (1561/1671)	91.1% (3325/3650)
BAC	98.6%	91.6%	90.2%
ROC-AUC	0.999	0.945	0.938

**Table 6:** Comparison of CNN3-CC-ECG performance to other methods for Sh/NSh rhythm detection during CPR. The results are reported exactly as published in studies.

Study	Method	Test Data	Se %	Sp %	BAC %
de Gauna et al., 2008 [29]	CPR suppression via Kalman filter with reference channel based on ECG Subsequent ECG analysis via standard AED shock advisory algorithm Input information: filtered CC-ECG	Analysis duration: 9.6–14.4 s Database: CC-ECG from real OHCA Test dataset: Independent - 131 Sh samples - 197 OR, 150 Asystole samples	90.1	80.4	85.3
Li et al., 2008 [40]	Wavelet transform and cross-correlation for ECG and CC morphology estimation. Evaluation of pattern differences. Input information: raw CC-ECG	Analysis duration: 10 s Database: CC-ECG from real OHCA Test dataset: Independent - 1256 Sh samples - 923 OR, 41 Asystole samples	93.3	88.6	91.0
Krasteva et al., 2010 [38]	Time–frequency techniques for ECG and CC morphology estimation. ECG signal reconstruction by subtraction of CC patterns. Input information: raw CC-ECG	Analysis duration: 10 s Database: CC-ECG from real OHCA Test dataset: Independent - 172 Sh samples - 371 OR, 330 Asystole samples	90.1	86.1	88.1
Issasi et al., 2020 [47]	CPR suppression via Recursive Least Squares filter with reference from a sternal CPR assist pad with an accelerometer. Subsequent ECG analysis via CNN classifier with three convolutional blocks and two fully connected layers. Input information: filtered CC-ECG	Analysis duration: 9 s Database: CC-ECG from real OHCA Test dataset: Not independent 5-fold cross-validation - 586 Sh samples - 1541 OR, 1192 Asystole samples	95.8	96.1	96.0
Issasi et al., 2020 [48]	CPR suppression via Recursive Least Squares filter with reference from a Load Distributing Band mechanical chest compression device. Subsequent ECG analysis via CNN classifier with three convolutional blocks and two fully connected layers Input information: filtered CC-ECG	Analysis duration: 8 s Database: CC-ECG from real OHCA Test dataset: Not independent Database split at 80/20% for training/validation - 780 Sh samples - 2644 OR + Asystole samples Median performance of 100 random repetitions reported	92.2	96.6	94.4
Hajeb-M et al., 2021 [49]	Hybrid DNN architecture: convolutional layers, residual blocks and bidirectional LSTM layers Input information: time and frequency domain ECG representations	Analysis duration: 8 s Database: Artificially mixed CC artifacts (OHCA Asystole) and clean-ECG (Holter) with fixed SNR = −3 dB. Test dataset: Not independent 4-fold cross-validation: - 3216 Sh rhythms - 6768 OR, missing Asystoles	94.2	86.1	90.1
This study	Fully convolutional DNN with three convolutional blocks, GMP and DENSE layer Input information: raw CC-ECG	Analysis duration: 10 s Database: CC-ECG from real OHCA Test dataset: Independent - 301 VF samples - 1640 OR, 3650 Asystole samples	89.0	91.3	90.2

## Data Availability

Restrictions apply to the availability of these data. Data were obtained from a third party (Schiller Médical SAS, Wissembourg, France) and are available on request from the corresponding author with the permission of the third party.
